# Ethnic disparities in demographic, clinicopathologic and biological behaviours and prognosis of gastric cancer in northwest China

**DOI:** 10.1002/cam4.3551

**Published:** 2020-10-20

**Authors:** Juan Cao, Jing Chen, Qinghua Zhang, Jing Wu, Wenfan Wang, Xiaoxu Zhang, Dan Zhao, Qian Zhang, Wenjun Yang, Zhiqiang Chen

**Affiliations:** ^1^ Medical Research Center Union Shenzhen Hospital Huazhong University of Science and Technology Shenzhen China; ^2^ Key Laboratory of Fertility Preservation and Maintenance (Ministry of Education) Ningxia Medical University Yinchuan China; ^3^ Department of Neurosurgery Union Shenzhen Hospital Huazhong University of Science and Technology Shenzhen China; ^4^ Department of Gastroenterology the General Hospital Ningxia Medical University Yinchuan China; ^5^ Department of Radiology the General Hospital Ningxia Medical University Yinchuan China

**Keywords:** EGFR, ethnic disparity, gastric cancer, Ki67, prognosis, VEGF

## Abstract

This retrospective study aimed to investigate ethnic disparities in demographic, clinicopathologic, and biological behaviours of gastric cancer (GC) in a high GC incidence area of China. There were 5022 GC patients, including 3987 Han (79.4%) and 987 Hui (14.4%) patients from Northwest China. All patient data were retrieved from 2009 to 2017. Median survival was estimated using the Kaplan‐Meier method and compared using the log‐rank test. A Cox proportional hazards model was used to assess the impact of covariates. Similarly, low 5‐year OS rates were observed in both the Hui and Han groups (23.8% and 24.2% respectively). Hui patients with stage T1 or N0 or with tumours <5 cm had 2.144‐fold, 1.426‐fold and 1.305‐fold increased risks of poor prognosis compared with Han patients with these characteristics respectively (all *p* < 0.05). Further, Hui patients had 1.265‐fold, 1.364‐fold and 1.401‐fold increased risks of poor prognosis compared with Han patients among those with high expression of Ki67, EGFR and VEGF respectively (all *p* < 0.05). There are ethnic disparities in the prognosis of GC patients in Northwest China. Understanding the effects of ethnicity on GC will guide reasonable evaluations of prognosis and future interventions to equalise access to high‐quality care for GC patients of different ethnicities in China.

## Graphical abstract

To our knowledge, this is the first study to examine the ethnic disparity between two main Chinese ethnic groups among 5022 gastric cancer (GC) patients from one of the highest GC incidence areas of China; the first study to assess the relevant biologic markers associated with GC prognosis between two Chinese ethnic groups. This knowledge can provide important information to both physicians and public health experts serving these populations to improve treatment and outcomes.

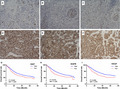

## 1 INTRODUCTION

Accumulating data suggest that geographical factors, such as ethnic density (the proportion of ethnic minority populations in a geographic area), and geographical segregation may be related to various cancer‐associated outcomes.^1^, ^2^ Therefore, identifying ethnic disparities and establishing ethnic survival models may offer a variety of social benefits, including greater availability of social support and community resources.

Gastric cancer (GC) has been prevalent in China for decades, with half of the world deaths (estimated 390,182 in 2018) occurring in China.^3^ Despite improvements in endoscopic, surgical and systemic treatments, the overall survival (OS) rates and prognosis of GC patients are still dismal. The 5‐year OS rate for GC is above 50% in Japan and Korea but only approximately 20% in China.^4^, ^5^, ^6^ Factors including older age, advanced TNM stage, poor differentiation and lymphatic metastasis have all been shown to be unfavourable for the prognosis of GC patients among different racial or ethnic groups.^7^, ^8^, ^9^ Some other factors, including environmental, as well as genetic background due to diversity in race/ethnicity, were also indicated to affect an individual's cancer spectrum. Two recent studies specifically indicated that race and ethnicity diversity played an important role in the prognosis of patients with GC.^10^, ^11^ Asian or Pacific Islander (API) GC patients were found to have a 1.262‐fold increased risk of poor prognosis compared to non‐API GC patients. Similarly, American Indian/Alaskan Natives have a 2.215‐fold increased risk of poor prognosis compared to white patients, who had a survival similar to that of API patients.^11^ Although ethnic differences in survival may partly be explained by inconsistent exposure to GC‐associated risk factors, such as unique clinical features, elevated rates of *Helicobacter pylori* infection, inappropriate treatment or cultural differences in lifestyle,^12^, ^13^, ^14^ there have been too few studies of ethnic density, especially among Asian ethnicities, to draw any meaningful conclusions with respect to cancer mortality. China has 56 officially recognised ethnicities and is becoming more ethnically diverse, although many ethnic minorities are geographically segregated in certain areas.^15^ To more fully understand the distinct contextual and compositional effects associated with different ethnicities, better data on risk factors, including relevant biological markers of cancer risk or outcomes, must be collected.^16^

In this study, patients with GC from two Chinese ethnic groups, namely Han and Hui, were retrospectively selected from one of the highest GC incidence areas: the Ningxia Hui Autonomous Region (hereafter called Ningxia), located on the Loess Plateau in Northwest China. Data from 5022 GC patients (3987 Han patients and 987 Hui patients) were reviewed, and their demographic, clinicopathologic and biological features, including Ki67, VEGF and EGFR were compared. These three cancer‐related biomarkers are routinely tested indexes and are clinically used to determine the degree of malignancy of GC and/or evaluate the prognosis of patients with GC.^17^, ^18^ To the best of our knowledge, this is the first study to examine the ethnic disparity in outcomes among GC patients between two main Chinese ethnic groups and evaluate prognosis and future interventions to equalise access to high‐quality care for GC patients of different ethnicities.

## 2 MATERIALS AND METHODS

### 2.1 Study population and data collection

A total of 5022 patients diagnosed with GC in the General Hospital of Ningxia Medical University, Ningxia, Northwest China from January 2009 to December 2017 were recruited for this retrospective study. Diagnosis was based on endoscopy and confirmatory histopathology. First, demographic data, including age at admission, sex, ethnicity, alcohol consumption and cigarette smoking, were collected from medical records. Those subjects who had three times or more alcoholic drinks a week for more than 6 months were defined as alcohol drinkers, and those who smoked one cigarette per day for more than 1 year were defined as smokers. Second, clinicopathologic characteristics, including T stage, N stage, M stage, tumour size, tumour location, Borrmann's type, Lauren's type, clinical staging and radical gastrectomy were further obtained. Patients with other previous cancers were excluded. T stage, N stage and M stage were assessed based on the 7th TNM stage system, and the clinical staging was assessed based on the TNM stage.

All procedures performed in studies involving human participants were in accordance with the ethical standards of the institutional and/or national research committee and with the 1964 Helsinki declaration and its later amendments or comparable ethical standards. The study was approved by the Ethical Committee of Ningxia Medical University prior to conducting the study. All the patients provided written informed consent for the review and use of their medical records according to the regulations.

### 2.2 Follow‐up

The information about the survival status, death time and cause of death was collected for all eligible GC patients from the hospital database, medical records or by telephone calling or text messages to patients or their family members. All patients were followed up through January 2019. Patients who were followed from treatment initiation to the date of death due to GC were considered as reaching the end point. Those patients who still cannot be contacted after three attempts or were noticed to be with wrong phone numbers were regarded as lost to follow‐up. Those who were alive, were lost to follow‐up or died from other diseases by the end of data collection were censored. The survival data were available for 3872 of 5022 GC patients (77.1%). The OS duration was calculated from treatment initiation data to either the date of death due to GC or the last follow‐up date.

### 2.3 Immunohistochemical analysis

For further immunohistochemical analysis, all the biopsy specimens were deparaffinised and serially sectioned into 4‐μm thick sections. The expression of Ki67, VEGF and EGFR in GC tissues was examined using the S‐P immunohistochemical method. Briefly, after routine deparaffinisation and rehydration, slides were treated with 1% hydrogen dioxide and then heated in EDTA (pH 8.0) for antigen retrieval. Following blocking in 10% goat serum, tissue sections were then incubated with mouse antibodies against human Ki67, VEGF and EGFR (ZSGB‐BIO, Beijing, China) at 4°C overnight. After rinsing, sections were subsequently incubated with goat anti‐mouse biotin‐conjugated IgG for 15 min and then with streptavidin‐peroxidase conjugate for 15 min. The signal was developed with diaminobenzidine, and slides were counterstained with 5% haematoxylin. The brown signals located in the cytoplasm represented positive staining for the respective proteins. Sections not treated with monoclonal antibody were used as negative controls. The expression of Ki67, VEGF and EGFR was assessed independently based on the proportion of positive cells by two pathologists who were blinded to the clinical data. The staining was scored on a scale from 0 to III as follows: 0, fewer than 10% of cells were stained; I, 10‐25% of cells were stained; II, 26‐50% of cells were stained; and III, >50% of cells were stained. A score of III was classified as high expression, whereas scores of 0‐II were considered low expression.^19^, ^20^, ^21^

### 2.4 Statistical analysis

Statistical analysis was performed using the Statistical Package for the Social Sciences 17.0 (SPSS, Inc., Microsoft, Chicago IL, USA). The distributions of the general demographic and clinicopathologic characteristics of the GC patients were analysed using the *χ* ^2^ test or Fisher's exact test for categorical variables, as appropriate. Cumulative survival rates were calculated from the treatment initiation date to the date of death or the last follow‐up. Median survival was estimated using the Kaplan‐Meier method and compared using the log‐rank test. Cox regression analysis was used to identify factors associated with survival in each population, and significant factors in the univariate analysis were included in the multivariate analysis. Relative risks were estimated by calculating hazard ratios (HRs) and 95% confidence intervals (CIs). Statistical significance was set at *p* < 0.05, and all statistical tests were two‐sided.

## 3 RESULTS

### 3.1 General demographic characteristics

The present study included 5022 GC patients aged 15–93 years diagnosed between 2009 and 2017 and followed through 2019. All patients’ characteristics are presented in Table S1. The median age of the patients was 61 years (interquartile range 53–68 years). A total of 3888 (77.4%) were men and 1134 (22.6%) were women, and the male‐to‐female ratio was 3.43:1. There were 2753 patients over the age of 60 years, accounting for 54.8% of all patients. Of these patients, 3,987 people were classified as Han (79.4%) and 987 were classified as Hui (14.4%) with the ratio of Han‐to‐Hui ratio being 4.04:1.

### 3.2 Ethnic differences in demographic and clinicopathologic features

A comparison of patients’ demographic and clinicopathologic features stratified by ethnicity is shown in Table 1. There were 2,047 (51.3%) patients over 60 years in Han group and 481 (48.7%) in Hui group. A total of 3067 (76.9%) male patients were in Han group and 782 (79.2%) males were in Hui group. Meanwhile, the ratios of male‐to‐female in Han and Hui groups were 3.33:1 and 3.81:1 respectively. There were no statistical differences in the distribution of age and sex between Han and Hui groups (all *p* > 0.05). Statistical differences were found between two ethnic groups in cigarette smoking, alcohol consumption, occupation, TNM stage, tumour location, Lauren's type, radical gastrectomy and radiotherapy (all *p* < 0.05). Hui patients were more likely than Han patients to be nonsmokers, nondrinkers and farmers. They were also more likely to present at a more advanced T stage, N stage and clinical staging. Compared with Han patients, Hui patients were less likely to receive radical gastrectomy or radiotherapy.

**TABLE 1:** Demographic, clinicopathologic and biologic variables of the Han and Hui GC patients

Variables	Total	Han (*n* = 3987)	Hui (*n* = 987)	*χ*2	*p*
Age					
≤60	2446	1940 (48.7)	506 (51.3)	2.154	0.142
>60	2528	2047 (51.3)	481 (48.7)		
Sex					
Male	3849	3067 (76.9)	782 (79.2)	2.402	0.121
Female	1125	920 (23.1)	205 (20.8)		
Cigarette smoking					
Yes	2129	1893 (47.8)	236 (24.0)	181.183	**<0.001**
No	2818	2071 (52.2)	747 (76.0)		
Alcohol drinking					
Yes	1036	986 (24.9)	50 (5.1)	186.683	**<0.001**
No	3906	2973 (75.1)	933 (94.9)		
Occupation					
Peasant	2267	1737 (43.6)	530 (53.8)	44.867	**<0.001**
Worker	1234	1051 (26.4)	183 (18.6)		
Other	350	301 (7.5)	49 (5.0)		
Unemployed	1122	898 (22.5)	224 (22.7)		
Blood type					
A	1329	1062 (29.8)	267 (30.4)	1.700	0.637
B	1368	1101 (30.9)	267 (30.4)		
AB	427	352 (9.9)	75 (8.6)		
O	1319	1051 (29.5)	268 (30.6)		
Rh					
Positive	3337	2657 (99.7)	680 (99.3)	2.603	0.157
Negative	13	8 (0.3)	5 (0.7)		
T					
T1	685	580 (17.5)	105 (13.1)	12.185	**0.007**
T2	514	422 (12.8)	92 (11.5)		
T3	588	473 (14.3)	115 (14.3)		
T4	2323	1833 (55.4)	490 (61.1)		
N					
N0	1539	1264 (41.3)	275 (37.2)	7.949	**0.047**
N1	909	733 (23.9)	176 (23.8)		
N2	768	615 (20.1)	153 (20.7)		
N3	585	449 (14.7)	136 (18.4)		
M					
M0	3627	2940 (87.4)	687 (85.4)	2.120	0.145
M1	542	425 (12.6)	117 (14.6)		
Tumour size (cm)					
<5	1801	1477 (50.8)	324 (48.3)	1.331	0.249
≥5	1780	1433 (49.2)	347 (51.7)		
Tumour location					
Upper	1028	786 (24.1)	242 (30.9)	16.148	**<0.001**
Middle	998	811 (24.8)	187 (23.9)		
Lower	2026	1671 (51.1)	355 (45.3)		
Differentiation					
High	370	310 (9.7)	60 (7.7)	4.768	0.092
Medium	1349	1096 (34.2)	253 (32.5)		
Low	2261	1795 (56.1)	466 (59.8)		
Lauren's type					
Intestinal	278	233 (48.8)	45 (40.2)	8.062	**0.018**
Diffuse	135	98 (20.5)	37 (33.0)		
Mixed	176	146 (30.6)	30 (26.8)		
Borrmann's type					
I	518	401 (12.4)	117 (14.8)	7.803	0.050
II	1294	1070 (33.1)	224 (28.4)		
III	1211	966 (29.9)	245 (31.1)		
IV	998	796 (24.6)	202 (25.6)		
Clinical staging					
I	697	585 (23.6)	112 (17.8)	16.660	**0.001**
II	631	518 (20.9)	113 (18.0)		
III	1234	956 (38.6)	278 (44.2)		
IV	542	416 (16.8)	126 (20.0)		
Pathological diagnosis					
Glandular cancer	4006	3221 (91.7)	785 (92.5)	3.748	0.290
Mucinous adenocarcinoma	90	71 (2.0)	19 (2.2)		
Signet‐ring cell carcinoma	152	121 (3.4)	31 (3.7)		
Other	112	98 (2.8)	14 (1.6)		
Radical gastrectomy					
Yes	3366	2724 (68.3)	642 (65.0)	3.924	**0.048**
No	1607	1262 (31.7)	345 (35.0)		
Chemotherapy					
Yes	1282	1015 (44.2)	267 (45.9)	0.499	0.480
No	1594	1279 (55.8)	315 (54.1)		
Radiotherapy					
Yes	123	107 (5.4)	16 (3.2)	3.953	**0.047**
No	2367	1885 (94.6)	482 (96.8)		
Palliative					
Yes	460	377 (9.5)	83 (8.4)	1.037	0.309
No	4513	3609 (90.5)	904 (91.6)		
Hp					
Positive	345	285 (33.6)	60 (32.1)	0.168	0.682
Negative	689	562 (66.4)	127 (67.9)		
Ki67					
Low‐expression	752	608 (35.5)	144 (33.3)	0.734	0.392
High‐expression	1391	1103 (64.5)	288 (66.7)		
VEGF					
Low‐expression	492	397 (28.1)	95 (25.7)	0.874	0.350
High‐expression	1290	1015 (71.9)	275 (74.2)		
EGFR					
Low‐expression	513	398 (28.1)	115 (31.2)	1.373	0.241
High‐expression	1274	1020 (71.9)	254 (68.8)		

Abbreviations: GC, gastric cancer.

Bold values represent the *P* values were below 0.05.

### 3.3 Survival and prognostic factors for OS between two ethnic groups

The median follow‐up time was 89.0 months. A total of 1734 (34.5%) GC patients died during the study period, and the overall median survival time was 74.0 months. No significant difference in OS was found between the two ethnic groups, and the 1‐year, 3‐year and 5‐year OS rates were 70.9%, 47.7% and 24.2%, respectively, for Han patients and 68.1%, 46.2% and 23.8%, respectively, for Hui patients, as shown in Table S2.

**Figure 1:**
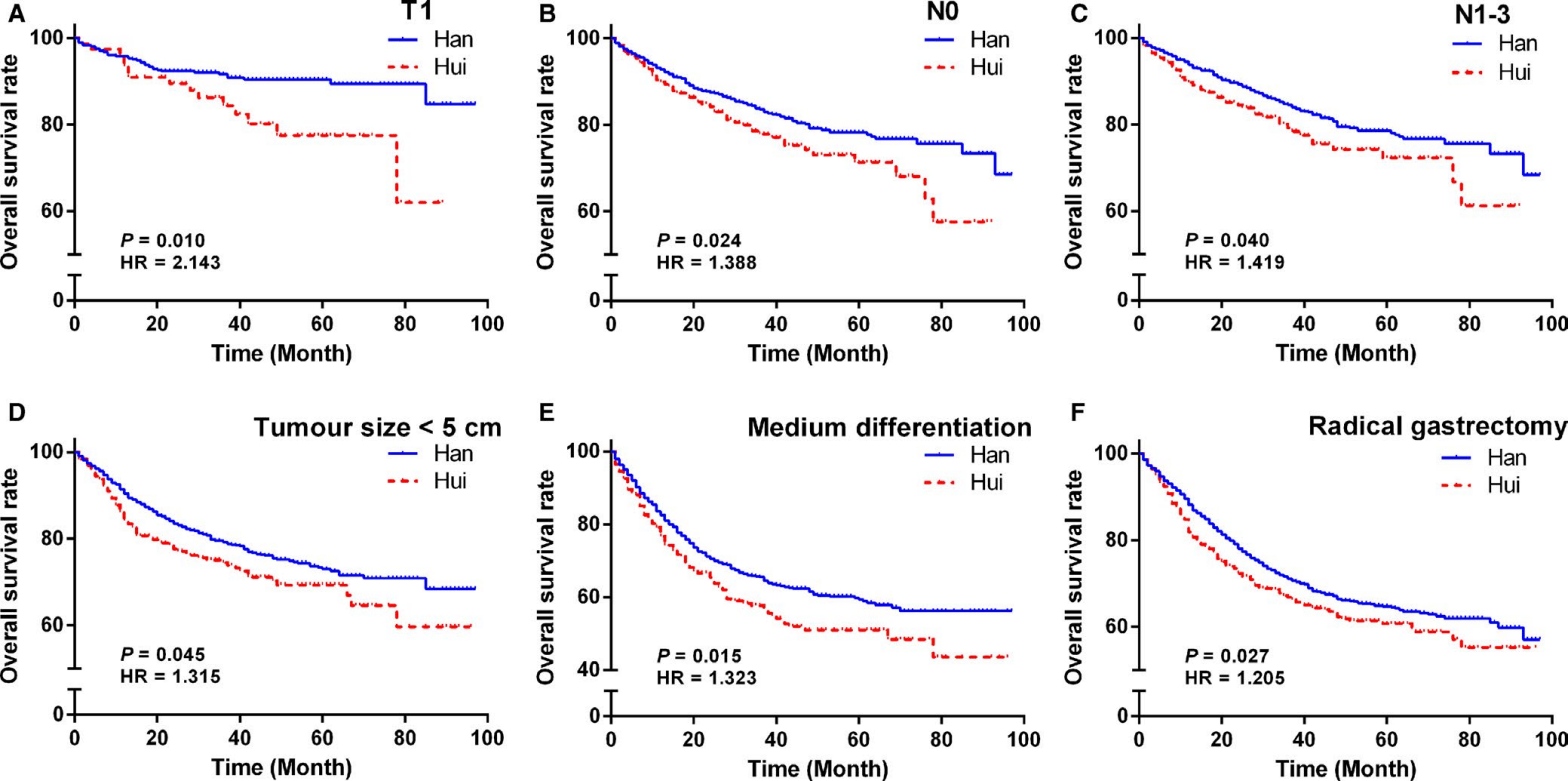
Survival curves of clinicopathologic features between Han and Hui patients with GC. (A–F) The survival curves of T1 (A), N0 (B), N1‐3 (C), tumour size below 5 cm (D), medium differentiation (E) and radical gastrectomy (F) between Hui and Han GC patients

**Figure 2:**
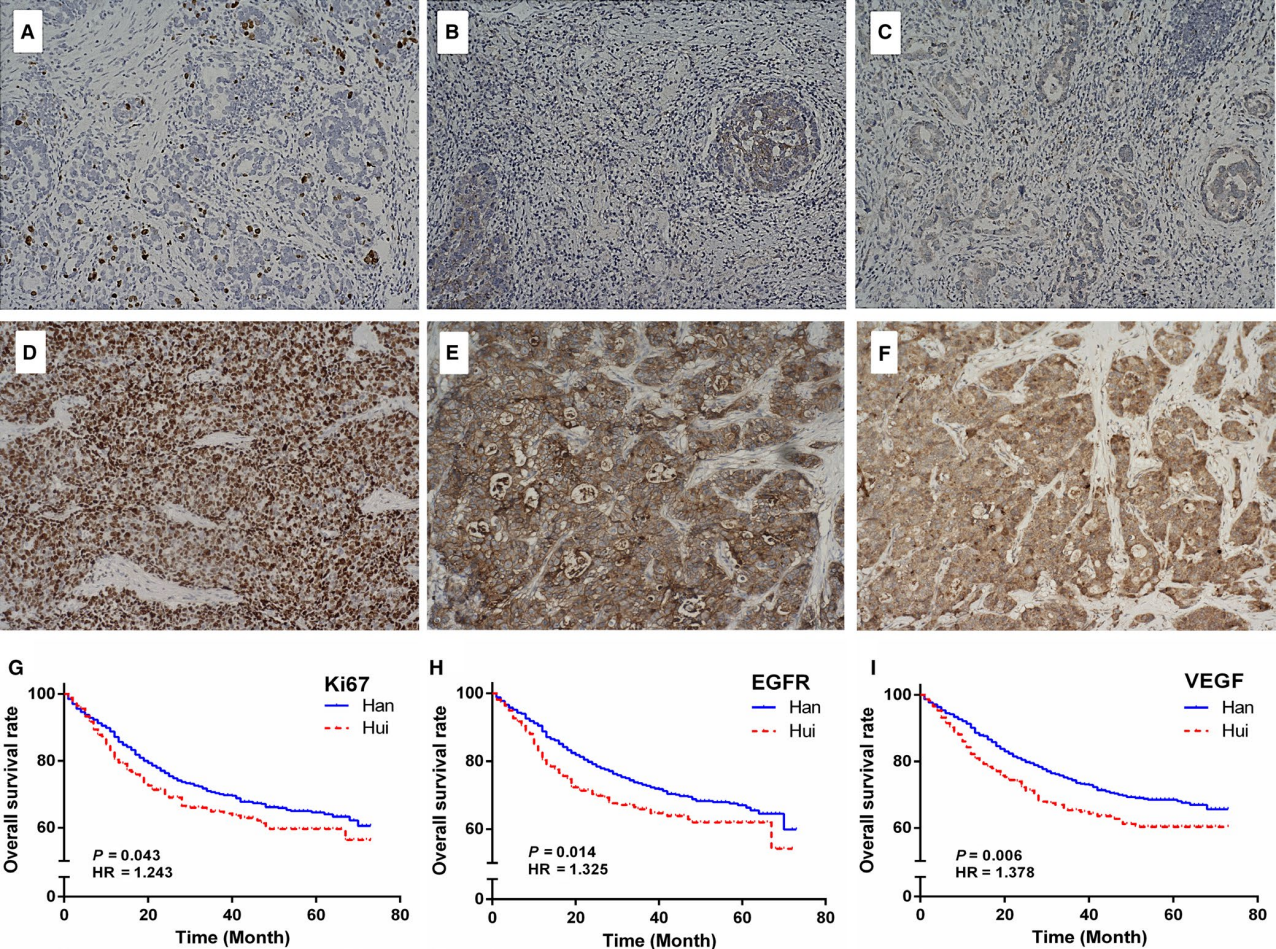
The immunohistochemistry staining of Ki67, EGFR and VEGF and the survival curves between Hui and Han GC patients. (A–F) Immunohistochemistry staining of Ki67, EGFR and VEGF in GC tissues and the brown signals represent positive staining: Low‐expression of Ki67 (A), EGFR (B) and VEGF (C). High‐expression of Ki67 (D), EGFR (E) and VEGF (F) (×200). (G‐I) The survival curves of Ki67 (G), EGFR (H) and VEGF (I) between Hui and Han GC patients

Univariate log‐rank analysis identified age, T stage, N stage, M stage, tumour size, tumour location, differentiation, Borrmann's type, clinical staging, radical gastrectomy and palliative status as significant prognostic factors for long‐term OS among both Han and Hui GC patients (all *p* < 0.05). Among patients with high expression of Ki67, EGFR or VEGF, the prognosis of Hui patients was much worse than that of Han patients (all *p* < 0.05). The details are shown in Table 2, Table S3, Figures 1, and 2.

**TABLE 2:** Univariate analysis of factors associated with mortality among the Han and Hui GC patients

Variables	Total	Han (*n* = 3987)		Hui (*n* = 987)		Pa
		Mortality (%)	*p*	Mortality (%)	*p*	
Age (years)			**<0.001**		**<0.001**	0.287
≤60	2446	488 (35.6)		140 (38.3)		0.373
>60	2528	805 (49.9)		207 (51.4)		0.517
Sex			0.334		0.804	0.355
Male	3888	1018 (43.9)		273 (44.8)		0.468
Female	1134	275 (41.3)		74 (46.2)		0.540
Cigarette smoking			0.357		0.949	0.234
Yes	2151	630 (44.5)		81 (45.8)		0.724
No	2844	646 (41.7)		264 (44.8)		0.237
Alcohol drinking			0.728		0.507	0.333
Yes	1047	319 (42.2)		19 (51.4)		0.359
No	3943	955 (43.3)		326 (44.7)		0.442
Occupation			0.926		0.105	0.381
Peasant	2278	590 (44.6)		186 (45.1)		0.710
Worker	1249	348 (42.9)		80 (51.9)		**0.031**
Other	355	64 (46.0)		8 (33.3)		0.226
Unemployed	1139	291 (40.9)		72 (40.4)		0.759
Blood type			0.488		0.623	0.217
A	1344	337 (43.3)		88 (42.3)		0.575
B	1388	330 (40.3)		85 (42.5)		0.697
AB	428	114 (42.9)		28 (46.7)		0.061
O	1327	312 (40.7)		98 (47.1)		0.662
Rh			0.819		0.852	0.064
Positive	3380	886 (38.7)		251 (42.7)		0.065
Negative	14	2 (28.6)		2 (40.0)		0.817
T			**<0.001**		**<0.001**	0.655
T1	691	38 (9.3)		15 (19.5)		**0.010**
T2	520	60 (19.7)		19 (28.8)		0.057
T3	592	143 (42.9)		31 (37.8)		0.461
T4	2343	732 (53.4)		209 (53.2)		0.831
N			**<0.001**		**<0.001**	0.334
N0	1553	170 (19.1)		52 (26.0)		**0.024**
N1	914	215 (42.4)		59 (45.0)		0.640
N2	776	234 (50.3)		59 (48.8)		0.618
N3	588	216 (58.2)		67 (57.3)		0.962
M			**<0.001**		**<0.001**	0.362
M0	3657	754 (35.4)		206 (39.4)		0.102
M1	548	239 (72.6)		66 (68.7)		0.336
Tumour size (cm)			**<0.001**		**<0.001**	0.320
<5	1817	251 (22.4)		72 (28.5)		**0.045**
≥5	1789	485 (50.3)		125 (49.2)		0.859
Tumour location			**<0.001**		**0.002**	0.619
Upper	1038	304 (47.0)		101 (50.2)		0.285
Middle	1010	244 (39.1)		46 (32.4)		0.107
Lower	2044	434 (36.8)		110 (41.2)		0.328
Differentiation			**<0.001**		**0.015**	0.319
High	375	70 (29.7)		10 (21.3)		0.903
Medium	1359	316 (36.8)		95 (46.6)		**0.015**
Low	2279	599 (47.7)		163 (46.8)		0.288
Lauren's type			0.089		0.490	0.394
Intestinal	282	51 (24.6)		10 (27.8)		0.614
Diffuse	135	29 (34.1)		12 (34.3)		0.796
Mixed	179	41 (32.3)		12 (41.4)		0.474
Borrmann's type			**<0.001**		**<0.001**	0.294
I	526	107 (32.8)		25 (36.9)		0.397
II	1299	217 (34.3)		55 (38.2)		0.432
III	1222	304 (42.0)		98 (49.2)		0.071
IV	1008	333 (50.0)		85 (50.3)		0.912
Pathological diagnosis			0.155		0.155	0.193
Adenocarcinoma	4039	981 (41.7)		276 (45.5)		0.071
Mucinous adenocarcinoma	91	29 (51.8)		7 (50.0)		0.736
Signet‐ring cell carcinoma	155	42 (42.9)		8 (32.0)		0.272
Other	114	29 (34.9)		2 (18.2)		0.239
Clinical staging			**<0.001**		**<0.001**	0.532
I	706	59 (11.4)		13 (13.1)		0.670
II	638	119 (26.2)		30 (30.9)		0.172
III	1248	386 (46.6)		124 (50.0)		0.435
IV	548	238 (70.0)		69 (65.1)		0.374
Radical gastrectomy			**<0.001**		**<0.001**	0.990
Yes	3390	617 (31.3)		177 (36.1)		**0.027**
No	1631	676 (67.7)		170 (60.9)		**0.044**
Chemotherapy			0.805		0.344	0.371
Yes	1295	340 (39.7)		90 (39.6)		0.886
No	1617	451 (41.1)		120 (44.3)		0.284
Radiotherapy			0.187		0.059	0.332
Yes	124	47 (50.0)		11 (68.7)		0.140
No	2398	651 (40.8)		172 (42.4)		0.503
Palliative			**<0.001**		**<0.001**	0.350
Yes	466	193 (76.3)		42 (67.7)		0.260
No	4555	1100 (40.3)		305 (43.1)		0.143
Hp			**0.006**		0.103	0.079
Positive	708	160 (47.5)		41 (47.1)		0.949
Negative	197	64 (34.8)		13 (31.0)		0.644
Ki67			0.164		0.316	**0.023**
Low‐expression	756	149 (28.1)		41 (32.8)		0.290
High‐expression	1410	302 (31.4)		96 (37.4)		**0.043**
VEGF			0.390		0.407	**0.012**
Low‐expression	497	109 (30.5)		27 (31.4)		0.770
High‐expression	1302	253 (28.9)		89 (37.2)		**0.006**
EGFR			0.682		0.390	**0.012**
Low‐expression	515	103 (29.9)		35 (34.0)		0.407
High‐expression	1289	261 (29.2)		81 (36.7)		**0.014**

Abbreviations: GC, gastric cancer; P for intra‐group univariate survival analysis; Pa for inter‐group univariate survival analysis.

Bold values represent the *P* values were below 0.05.

Given that the clinical staging was assessed based on the T stage, N stage and M stage, the collinearity diagnosis was used. The results showed that the tolerance value was 0.097 and the Variance Inflation Factor (VIF) was 10.304 in the clinical staging, which demonstrated that collinearity exists between clinical staging and T, N and M covariates. Therefore, all variables with statistical significance in the univariate analyses except for the clinical staging were enrolled in the Cox proportional hazards model. In addition, we have compared the distributions of the missing data in each covariate between Han and Hui groups, which showed no statistical significances. The Cox proportional hazards model revealed that four variables (age, T stage, N stage and tumour size ) were significant independent prognostic factors in both Han and Hui patients; M stage was also an independent predictor in Han patients, while low differentiation was an independent predictor in Hui patients. The model also showed that patients who received radical gastrectomy had a longer OS than patients who did not. The HRs in both Han and Hui were significantly higher for older age (1.030 vs 1.029), tumour size greater than 5 cm (1.330 vs 1.399), advanced T4 stage (4.148 vs 2.084) and advanced N3 stage (2.727 vs 1.962) (all *p* < 0.05) (Table 3).

**TABLE 3:** Multivariate survival analysis of factors associated with OS among the Han and Hui GC patients

Variables	Han		Hui	
	HR (95% CI)	*p*	HR (95% CI)	*p*
Age (years)	1.030 (1.020, 1.040)	**<0.001**	1.029 (1.009, 1.049)	**0.004**
T				
T1	1.000		1.000	
T2	1.952 (1.077, 3.539)	**0.027**	1.400 (0.674, 2.908)	0.367
T3	3.663 (2.089, 6.421)	**<0.001**	1.626 (0.826, 3.198)	0.159
T4	4.148 (2.428, 7.087)	**<0.001**	2.084 (1.132, 3.838)	**0.018**
N				
N0	1.000		1.000	
N1	1.606 (1.211, 2.130)	**0.001**	1.390 (0.922, 2.098)	0.116
N2	1.816 (1.366, 2.413)	**<0.001**	1.479 (0.971, 2.254)	0.068
N3	2.727 (2.038, 3.649)	**<0.001**	1.962 (1.290, 2.985)	**0.002**
M				
M0	1.000		1.000	
M1	1.975 (1.383, 2.820)	**<0.001**	1.347 (0.880, 2.061)	0.170
Tumour size (cm)				
<5	1.000		1.000	
≥5	1.330 (1.089, 1.623)	**0.005**	1.399 (1.014, 1.930)	**0.041**
Tumour location				
Upper	1.000		1.000	
Middle	1.045 (0.836, 1.306)	0.698	1.212 (0.822, 1.786)	0.332
Lower	0.949 (0.755, 1.194)	0.655	0.664 (0.405, 1.088)	0.104
Differentiation				
High	1.000		1.000	
Medium	0.906 (0.608, 1.349)	0.626	2.287 (0.904, 5.786)	0.081
Low	1.360 (0.924, 2.004)	0.119	2.570 (1.019, 6.482)	**0.046**
Borrmann's type				
I	1.000		1.000	
II	1.329 (0.971, 1.820)	0.076	1.362 (0.725, 2.559)	0.336
III	1.254 (0.921, 1.709)	0.151	1.674 (0.911, 3.077)	0.097
IV	0.835 (0.602, 1.158)	0.280	1.251 (0.644, 2.431)	0.509
Radical gastrectomy				
No	1.000		1.000	
Yes	0.371 (0.275, 0.502)	**<0.001**	0.557 (0.389, 0.798)	**0.001**

Abbreviations: GC, gastric cancer; OS, overall survival; HR, hard ratio; CI, confidence interval.

Bold values represent the *P* values were below 0.05.

## 4 DISCUSSION

In China, there are 56 ethnic groups, with Han people having the highest population (92%) and Hui people having the third highest population (0.7%) of 55 minority populations. Most Hui people are Muslims and descendants of soldiers, merchants and political emissaries from Arabia and Persia.^22^ Approximately 2.2 million Hui people, who are characterised by religious purity, group identity and different living habits,^23^, ^24^ live in the Ningxia region of Northwest China, which has the highest incidence and mortality of GC. In this study, compared with Han patients, Hui patients were less likely to smoke or consume alcohol, but they were more likely to present at a more advanced T stage, N stage and clinical staging. Although no statistically significant difference in OS was found between the two ethnic groups, the 1‐year, 3‐year and 5‐year OS of both Hui and Han patients from northwestern China (70.3%, 47.5% and 24.1% respectively) were worse than those patients from southern China (86.2%, 52.5% and 36.0% respectively).^25^ Besides, the 1‐year, 3‐year and 5‐year OS rates of Hui patients (68.1%, 46.2% and 23.8% respectively) were all shown to be lower than the corresponding OS rates of Han patients (70.9%, 47.7% and 24.2%). Further, Hui patients at an early stage, such as those with T1 or N0 disease, and those with tumours <5 cm, had 2.144‐fold, 1.426‐fold and 1.305‐fold increased risks of poor prognosis compared with similar Han patients respectively.

The similar low OS rates of two ethnic groups and the survival disparities between Hui and Han people may be explained by several factors. First, Ningxia is one of the economically underdeveloped regions in China. The lagged education level, the limited medical resources and low income in this area may all contribute to the poor survival we observed in both the Hui and Han GC populations. Second, most Hui people live in rural mountain areas within a closed circle for historical reasons, which limits their acceptance of modern medical knowledge, especially the importance of health care and early treatment. Even worse, many Hui people, who are subsistence farmers, belong to the low‐income class and have difficulty affording medical expenses. Last, some Hui people, who are influenced by their religious beliefs, are unwilling to accept surgery or other treatments.

Ethnicity may also reflect genetic biological effects in addition to origin, history and cultural identity. An understanding of the biological and molecular basis of GC will enable oncologic treatment or prognostic assessments to be tailored to the unique characteristics of individual tumours. Some reports have showed that high expression of Ki67, EGFR or VEGF was an unfavourable factor for GC prognosis;^18^, ^26^, ^27^ our findings showed that, among patients with high expression of Ki67, EGFR or VEGF, the prognosis of Hui patients was much worse than that of Han patients, which indicated that Hui patients exhibiting high expression levels of three cancer‐related genes seemed to be more susceptible to a poor prognosis. Although we do not know the underlying reasons for this phenomenon, several studies have demonstrated hereditary genetic diversity between Hui and Han populations. Li et al. systemically investigated the effects of exogenous additives on the genetic diversity of drug absorption, distribution, metabolism and excretion between the Han majority and Hui minority in Northwest China and reported significant differences between the Han and Hui populations.^28^ Our previous study also showed that the distribution of genetic variants in cancer‐related genes varies between Han and Hui people.^24^ These studies highlight that genetic background diversity in ethnicities, such as the Han and Hui populations, may affect an individual's cancer spectrum. These results have significant implications for assessing cancer susceptibility, sensitivity to treatments and prognosis among different ethnic groups.

The limitations of the present study need to be considered in the interpretation of the obtained results. All of the participants were enrolled from one region, and the subjects may not represent all GC patients in China. Therefore, further investigations with multiple populations warrant confirmation. Another limitation of this study is the lack of information on socioeconomic status, which may also affect the prognosis of GC patients.^29^

## 5 CONCLUSIONS

In conclusion, there are ethnic disparities in the survival status of Hui and Han GC patients in Northwest China, even after hundreds of years of living together. Hui GC patients were more likely to have a poorer prognosis than Han patients among those with early stage disease or high expression of Ki67, EGFR or VEGF. An understanding of the effects of ethnicity on GC will prompt public health experts and policy makers to identify, intervene and eventually alleviate the root causes of GC disparities.

## CONFLICT OF INTERESTS

The authors declared no potential conflict of interest.

## Supporting information

Supplementary Material

## Data Availability

Data available on request due to privacy/ethical restrictions.

## References

[1] Gomez SL , Shariff‐Marco S , DeRouen M , et al. The impact of neighborhood social and built environment factors across the cancer continuum: current research, methodological considerations, and future directions. Cancer. 2015;121(14):2314‐2330. 10.1002/cncr.29345 25847484 PMC4490083

[2] Landrine H , Corral I , Lee JGL , Efird JT , Hall MB , Bess JJ . Residential segregation and racial cancer disparities: a systematic review. J Racial Ethn Health Disparities. 2017;4(6):1195‐1205. 10.1007/s40615-016-0326-9 28039602

[3] Bray F , Ferlay J , Soerjomataram I , Siegel RL , Torre LA , Jemal A . Global cancer statistics 2018: GLOBOCAN estimates of incidence and mortality worldwide for 36 cancers in 185 countries. CA Cancer J Clin. 2018;68(6):394‐424. 10.3322/caac.21492 30207593

[4] Zhao L , Huang H , Zhao D , et al. Clinicopathological characteristics and prognosis of proximal and distal gastric cancer during 1997–2017 in china national cancer center. J Oncol. 2019;2019:9784039 10.1155/2019/9784039 31312217 PMC6595386

[5] Gu H , Li D , Zhu H , et al. The prognostic efficacy and improvements of the 7th edition union for international cancer control tumor‐node‐metastasis classifications for chinese patients with gastric cancer: Results based on a retrospective three‐decade population study. Tumor Biol. 2017;39(3): 10.1177/1010428317694548 28351302

[6] Zuo TT , Zheng RS , Zeng HM , Zhang SW , Chen WQ . Epidemiology of gastric cancer in China. Clinical Oncology in China. 2017;44(01):52‐58. (In Chinese).

[7] Kattan MW , Karpeh MS , Mazumdar M , Brennan MF . 2Postoperative nomogram for disease‐specific survival after an R0 resection for gastric carcinoma. J Clin Oncol. 2003;21(19):3647‐3650. 10.1200/JCO.2003.01.240 14512396

[8] Eom BW , Ryu KW , Nam BH , et al. Survival nomogram for curatively resected korean gastric cancer patients: multicenter retrospective analysis with external validation. PLoS One. 2015;10(2):e0119671 10.1371/journal.pone.0119671 25723182 PMC4344235

[9] Wang W , Sun Z , Deng JY , Wang ZN , Zhou ZW , Liang H . Integration and analysis of associated data in surgical treatment of gastric cancer based on multicenter, high volume databases. Zhonghua Wei Chang Wai Ke Za Zhi. 2016;19(2):179‐185. (In Chinese). 26831882

[10] Hallowell BD , Endeshaw M , Senkomago V , Razzaghi H , McKenna MT , Saraiya M . Gastric cancer mortality rates among US and foreign‐born persons: United States 2005–2014. Gastric Cancer. 2019;22(5):1081‐1085. 10.1007/s10120-019-00944-w 30830640 PMC6697193

[11] Zhang G , Zhao X , Li J , et al. Racial disparities in stage‐specific gastric cancer: analysis of results from the Surveillance Epidemiology and End Results (SEER) program database. J Investig Med. 2017;65(6):991‐998. 10.1136/jim-2017-000413 28442533

[12] Luyimbazi D , Nelson RA , Choi AH , et al. Estimates of conditional survival in gastric cancer reveal a reduction of racial disparities with long‐term follow‐up. J Gastrointest Surg. 2015;19(2):251‐257. 10.1007/s11605-014-2688-9 25421357

[13] Liu J , Geng Q , Liu Z , et al. Development and external validation of a prognostic nomogram for gastric cancer using the national cancer registry. Oncotarget. 2016;7(24):35853‐35864. 10.18632/oncotarget.8221 27016409 PMC5094968

[14] Hester C , Yopp A , Polanco P , Mansour J , Wang S , Porembka M . Ethnic and racial disparities among young patients with noncardia gastric cancer. Ann Oncol. 2018;29(Suppl 5):v25 10.1093/annonc/mdy151.089

[15] Ethnic minorities in China [homepage on the Internet] . No date. Available from: (https://en.m.wikipedia.org/wiki/Ethnic_minorities_in_China).

[16] Fang CY , Tseng M Ethnic density and cancer: a review of the evidence. Cancer. 2018;124(9):1877‐1903. 10.1002/cncr.31177 29411868 PMC5920546

[17] Wieduwilt MJ , Moasser MM . The epidermal growth factor receptor family: Biology driving targeted therapeutics. Cell Mol Life Sci. 2008;65(10):1566‐1584. 10.1007/s00018-008-7440-8 18259690 PMC3060045

[18] Xiong DD , Zeng CM , Jiang L , Luo DZ , Chen G . Ki‐67/MKI67 as a predictive biomarker for clinical outcome in gastric cancer patients: an updated meta‐analysis and systematic review involving 53 studies and 7078 patients. J Cancer. 2019;10(22):5339‐5354. 10.7150/jca.30074 31632479 PMC6775696

[19] Nikiteas NI , Tzanakis N , Theodoropoulos G , et al. Vascular endothelial growth factor and endoglin (CD‐105) in gastric cancer. Gastric Cancer. 2007;10:12‐17. 10.1007/s10120-006-0401-8 17334712

[20] Huang GL , Chen SQ , Wang DY , et al. High Ki67 expression has prognostic value in surgically‐resected T3 gastric adenocarcinoma. Clin Lab. 2016;62:141‐153. 10.7754/Clin.Lab.2015.150610 27012044

[21] Wang D , Wang B , Wang R , et al. High expression of EGFR predicts poor survival in patients with resected T3 stage gastric adenocarcinoma and promotes cancer cell survival. Oncol Lett. 2017;13:3003‐3013. 10.3892/ol.2017.5827 28521408 PMC5431275

[22] Gladney DC . Muslim tombs and ethnic folklore: charters for Hui identity. J Asian Stud. 1987;46(3):495‐532.

[23] Xu S . Human population admixture in Asia. Genomics Inform. 2012;10(3):133‐144. 23166524 10.5808/GI.2012.10.3.133 PMC3492649

[24] Tian C , Chen Z , Ma X , et al. Comparison of genetic variants in cancer‐related genes between Chinese Hui and Han populations. PLoS One. 2015;10(12):e0145170. 26683024 10.1371/journal.pone.0145170 PMC4684198

[25] Fang L , Hu Y . Survival analysis of gastric cancer patients in Zhejiang province from 2011 to 2016[J]. Chinese Journal of Health Statistics. 2019;036(005):735‐736,739. (In Chinese).

[26] Nagatsuma AK , Aizawa M , Kuwata T , et al. Expression profiles of HER2, EGFR, MET and FGFR2 in a large cohort of patients with gastric adenocarcinoma. Gastric Cancer. 2015;18(2):227‐238. 10.1007/s10120-014-0360-4 24626858

[27] Kim MA , Lee HS , Lee HE , Jeon YK , Yang HK , Kim WH . EGFR in gastric carcinomas: prognostic significance of protein overexpression and high gene copy number. Histopathology. 2008;52(6):738‐746. 10.1111/j.1365-2559.2008.03021.x 18397279

[28] Li J , Zhang L , Zhou H , Stoneking M , Tang K . Global patterns of genetic diversity and signals of natural selection for human ADME genes. Hum Mol Genet. 2011;20(3):528‐540. 10.1093/hmg/ddq498 21081654

[29] Singh GK , Ahmedin J . Socioeconomic and Racial/Ethnic Disparities in Cancer Mortality, Incidence, and Survival in the United States, 1950–2014: Over Six Decades of Changing Patterns and Widening Inequalities. J Environ Public Health. 2017;2017:1‐19. 10.1155/2017/2819372 PMC5376950 28408935

