# Supplementary material for: Ethnic disparities in demographic, clinicopathologic and biological behaviours and prognosis of gastric cancer in northwest China
Source: Cancer Med. 2020 Oct 20;9(24):9353–64. doi: 10.1002/cam4.3551 (PMC7774720; doi:10.1002/cam4.3551)
Supplement: Supplementary file 1 — Supplementary Material [file CAM4-9-9353-s001.doc]

| Table S1. Demographic data of all subjects recruited in this study. | | | | | |
| --- | --- | --- | --- | --- | --- |
| Characteristics | | No.% | Characteristics | | No.% |
| Age | ≤60 | 2446 (48.7) | T | T1 | 691 (13.8) |
|  | >60 | 2528 (51.3) |  | T2 | 520 (10.4) |
| Sex | Male | 3888 (77.4) |  | T3 | 592 (11.8) |
|  | Female | 1134 (22.6) |  | T4 | 2343 (46.7) |
| Ethnicity | Han | 3987 (79.4) | N | N0 | 1553 (30.9) |
|  | Hui | 987 (19.7) |  | N1 | 914 (18.2) |
| Cigarette Smoking | Yes | 2151 (42.8) |  | N2 | 776 (15.5) |
|  | No | 2844 (56.6) |  | N3 | 588 (11.7) |
| Alcohol drinking | Yes | 1047 (20.8) | M | M0 | 3657 (72.8) |
|  | No | 3943 (78.5) |  | M1 | 548 (10.9) |
| Occupation | Peasant | 2278 (45.4) | Borrmann’s type | I | 526 (10.5) |
|  | Worker | 1249 (24.9) |  | II | 1299 (25.9) |
|  | Other | 355 (7.1) |  | III | 1222 (24.3) |
|  | Unemployed | 1139 (22.7) |  | IV | 1008 (20.1) |
| Blood type | A | 1344 (26.8) | Clinical staging | I | 706 (14.1) |
|  | B | 1387 (27.6) |  | II | 638 (12.7) |
|  | AB | 428 (8.5) |  | III | 1248 (24.9) |
|  | O | 1325 (26.4) |  | IV | 548 (10.9) |
| Rh | Positive | 3380 (67.3) | Tumour size | <5 | 1817 (36.2) |
|  | Negative | 14 (0.3) |  | ≥5 | 1789 (35.6) |
| Lauren’s type | Intestinal | 282 (5.6) | Differentiation | High | 375 (7.5) |
|  | Diffuse | 135 (2.7) |  | Medium | 1359 (27.1) |
|  | Mixed | 179 (3.6) |  | Low | 2279 (45.4) |
| Tumour location | Upper | 1038 (20.7) | Hp | Positive | 349 (6.9) |
|  | Middle | 1010 (20.1) |  | Negative | 695 (13.8) |
|  | Lower | 2044 40.7) | Chemotherapy | Yes | 1295 (25.8) |
| Radical gastrectomy | Yes | 3390 (67.5) |  | No | 1617 (32.2) |
|  | No | 1631 (32.5) | Radiotherapy | Yes | 124 (2.5) |
| Palliative | Yes | 466 (9.3) |  | No | 2398 (47.7) |
|  | No | 4555 (90.7) | Pathological diagnosis | Glandular cancer | 3806 (75.8) |
| Ki67 | Low-expression | 756 (15.1) |  | Mucinous adenocarcinoma | 67 (1.3) |
|  | High-expression | 1410 (28.1) |  | Signet-ring cell carcinoma | 171 (3.4) |
| EGFR | Low-expression | 515 (10.3) |  | Other | 160 (3.2) |
|  | High-expression | 1289 (25.7) |  |  |  |
| VEGF | Low-expression | 497 (9.9) |  |  |  |
|  | High-expression | 1302 (25.9) |  |  |  |

| Table S2. The overall survival of the Han and Hui GC patients. | | | | | | | | | | | |
| --- | --- | --- | --- | --- | --- | --- | --- | --- | --- | --- | --- |
| Group | Min | Max | Mean ± SD | Percentiles | | |  | Overall survival rate | | | |
|  |  |  |  | 25 (ms) | 50 (ms) | 75 (ms) |  | 1-Year (%) | 2-Year (%) | 3-Year (%) | 5-Year (%) |
| Han | 0.00 | 97.00 | 31.96 ± 22.70 | 14.00 | 28.00 | 48.00 |  | 70.9 | 57.8 | 47.7 | 24.2 |
| Hui | 0.00 | 96.00 | 31.60 ± 23.27 | 12.00 | 27.00 | 48.00 |  | 68.1 | 55.3 | 46.2 | 23.8 |
| Total | 0.00 | 97.00 | 31.86 ± 22.82 | 13.20 | 27.25 | 48.00 |  | 70.3 | 57.3 | 47.5 | 24.1 |
| GC, Gastric Cancer; ms,months | | | | | | | | | | | |

| Table S3. Univariate analysis of factors associated with mortality among the Han and Hui GC patients. | | | | | | |
| --- | --- | --- | --- | --- | --- | --- |
| Variables | Han (n= 3987) | | Hui (n= 987) | | HR (95%CI) | *Pa* |
|  | HR (95%CI) | *P* | HR (95%CI) | *P* |  |  |
| Age (years) |  |  |  |  |  |  |
| ≤60 | 1.000 |  | 1.000 |  | 1.089 (0.902, 1.314) | 0.376 |
| >60 | 1.616 (1.444, 1.808) | **<0.001** | 1.551 (1.251, 1.923) | **<0.001** | 1.051 (0.902, 1.225) | 0.521 |
| Sex |  |  |  |  |  |  |
| Female | 1.000 |  | 1.000 |  | 1.083 (0.838, 1.400) | 0.543 |
| Male | 1.067 (0.934, 1.219) | 0.339 | 1.033 (0.799, 1.336) | 0.805 | 1.050 (0.919, 1.200) | 0.472 |
| Cigarette smoking |  |  |  |  |  |  |
| No | 1.000 |  | 1.000 |  | 1.089 (0.944, 1.257) | 0.241 |
| Yes | 1.052 (0.943, 1.175) | 0.361 | 1.008 (0.786, 1.293) | 0.949 | 1.042 (0.827, 1.314) | 0.726 |
| Alcohol drinking |  |  |  |  |  |  |
| No | 1.000 |  | 1.000 |  | 1.050 (0.926, 1.191) | 0.446 |
| Yes | 0.978 (0.862, 1.110) | 0.730 | 1.168 (0.735, 1.855) | 0.511 | 1.239 (0.780, 1.969) | 0.364 |
| Occupation |  |  |  |  |  |  |
| Peasant | 1.000 |  | 1.000 |  | 1.032 (0.875, 1.216) | 0.712 |
| Worker | 0.960 (0.841, 1.096) | 0.543 | 1.217 (0.936, 1.582) | 0.142 | 1.303 (1.022, 1.662) | 0.033 |
| Other | 0.973 (0.752, 1.260) | 0.837 | 0.598 (0.294, 1.215) | 0.155 | 0.639 (0.306, 1.334) | 0.233 |
| Unemployed | 0.965 (0.839, 1.111) | 0.624 | 0.900 (0.685, 1.181) | 0.446 | 0.961 (0.742, 1.244) | 0.761 |
| Blood type |  |  |  |  |  |  |
| B | 1.000 |  | 1.000 |  | 1.070 (0.843, 1.358) | 0.577 |
| A | 1.111 (0.954, 1.293) | 0.175 | 0.986 (0.732, 1.328) | 0.925 | 0.955 (0.755, 1.207) | 0.699 |
| O | 1.010 (0.865, 1.179) | 0.898 | 1.168 (0.874, 1.562) | 0.294 | 1.240 (0.988, 1.556) | 0.063 |
| AB | 1.082 (0.874, 1.339) | 0.469 | 1.105 (0.721, 1.695) | 0.645 | 1.096 (0.724, 1.657) | 0.665 |
| Rh |  |  |  |  |  |  |
| Negative | 1.000 |  | 1.000 |  | 1.260 (0.176, 8.999) | 0.818 |
| Positive | 1.174 (0.293, 4.701) | 0.821 | 1.141 (0.284, 4.588) | 0.853 | 1.140 (0.991, 1.311) | 0.068 |
| T |  |  |  |  |  |  |
| T1 | 1.000 |  | 1.000 |  | 2.144 (1.179, 3.900) | **0.012** |
| T2 | 2.185 (1.455, 3.280) | **<0.001** | 1.661 (0.844, 3.269) | 0.142 | 1.641 (0.979, 2.751) | 0.060 |
| T3 | 5.302 (3.707, 7.582) | **<0.001** | 2.153 (1.162, 3.989) | **0.015** | 0.865 (0.587, 1.275) | 0.464 |
| T4 | 7.805 (5.631, 10.817) | **<0.001** | 3.585 (2.122, 6.056) | **<0.001** | 0.984 (0.843, 1.147) | 0.833 |
| N |  |  |  |  |  |  |
| N0 | 1.000 |  | 1.000 |  | 1.426 (1.045, 1.945) | **0.025** |
| N1 | 2.636 (2.156, 3.224) | **<0.001** | 1.977 (1.361, 2.871) | **<0.001** | 1.071 (0.803, 1.428) | 0.643 |
| N2 | 3.356 (2.754, 4.090) | **<0.001** | 2.177 (1.499, 3.162) | **<0.001** | 0.931 (0.699, 1.238) | 0.621 |
| N3 | 4.333 (3.541, 5.302) | **<0.001** | 3.021 (2.100, 4.346) | **<0.001** | 1.007 (0.765, 1.324) | 0.963 |
| M |  |  |  |  |  |  |
| M0 | 1.000 |  | 1.000 |  | 1.136 (0.974, 1.326) | 0.104 |
| M1 | 3.437 (2.967, 3.980) | **<0.001** | 2.641 (1.997, 3.492) | **<0.001** | 0.878 (0.668, 1.153) | 0.349 |
| Tumour size (cm) |  |  |  |  |  |  |
| <5 | 1.000 |  | 1.000 |  | 1.305 (1.004, 1.695) | **0.047** |
| ≥5 | 2.572 (2.208, 2.995) | **<0.001** | 1.915 (1.433, 2.560) | **<0.001** | 0.983 (0.807, 1.196) | 0.860 |
| Tumour location |  |  |  |  |  |  |
| Upper | 1.000 |  | 1.000 |  | 1.129 (0.902, 1.415) | 0.289 |
| Middle | 0.823 (0.695, 0.974) | **0.023** | 0.556 (0.392, 0.789) | **0.001** | 0.774 (0.565, 1.061) | 0.111 |
| Lower | 0.727 (0.628, 0.842) | **<0.001** | 0.705 (0.538, 0.925) | **0.012** | 1.109 (0.900, 1.367) | 0.331 |
| Differentiation |  |  |  |  |  |  |
| High | 1.000 |  | 1.000 |  | 0.701 (0.361, 1.360) | 0.293 |
| Medium | 1.296 (1.000, 1.679) | **<0.001** | 2.451 (1.294, 4.643) | **0.006** | 1.325 (1.053, 1.666) | **0.016** |
| Low | 1.742 (1.360, 2.231) | 0.050 | 2.445 (1.274, 4.691) | **0.007** | 0.989 (0.832, 1.176) | 0.904 |
| Lauren’s type |  |  |  |  |  |  |
| Intestinal | 1.000 |  | 1.000 |  | 1.190 (0.603, 2.346) | 0.616 |
| Diffuse | 1.430 (0.905, 2.258) | 0.125 | 1.365 (0.587, 3.176) | 0.470 | 1.092 (0.557, 2.141) | 0.798 |
| Mixed | 1.530 (1.014, 2.311) | **0.043** | 1.658 (0.712, 3.863) | 0.241 | 1.263 (0.662, 2.410) | 0.479 |
| Borrmann’s type |  |  |  |  |  |  |
| I | 1.000 |  | 1.000 |  | 0.829 (0.537, 1.282) | 0.400 |
| II | 1.090 (0.864, 1.375) | 0.466 | 1.451 (0.903, 2.330) | 0.124 | 1.125 (0.837, 1.513) | 0.435 |
| III | 1.467 (1.177, 1.829) | **0.001** | 2.151 (1.386, 3.339) | **0.001** | 1.231 (0.980, 1.546) | 0.074 |
| IV | 1.814 (1.459, 2.255) | **<0.001** | 2.156 (1.380, 3.368) | **0.001** | 0.987 (0.778, 1.252) | 0.913 |
| Pathological diagnosis |  |  |  |  |  |  |
| Adenocarcinoma | 1.000 |  | 1.000 |  | 1.130 (0.988, 1.291) | 0.074 |
| Mucinous adenocarcinoma | 1.794 (1.072, 3.003) | **0.026** | 3.794 (0.788, 18.273) | 0.096 | 0.869 (0.380, 1.989) | 0.741 |
| Signet-ring cell carcinoma | 1.294 (0.894, 1.872) | 0.172 | 3.409 (0.848, 13.702) | 0.084 | 0.658 (0.309, 1.402) | 0.278 |
| Other | 1.362 (0.849, 2.187) | 0.200 | 2.133 (0.453, 10.046) | 0.338 | 0.438 (0.104, 1.837) | 0.259 |
| Clinical staging |  |  |  |  |  |  |
| I | 1.000 |  | 1.000 |  | 1.139 (0.625, 2.077) | 0.671 |
| II | 2.370 (1.734, 3.238) | **<0.001** | 2.668 (1.392, 5.116) | **0.003** | 1.320 (0.884, 1.971) | 0.175 |
| III | 5.069 (3.853, 6.668) | **<0.001** | 4.739 (2.675, 8.394) | **<0.001** | 1.083 (0.885, 1.326) | 0.439 |
| IV | 10.661 (8.011, 14.189) | **<0.001** | 8.001 (4.420, 14.487) | **<0.001** | 0.888 (0.679, 1.161) | 0.385 |
| Radical gastrectomy |  |  |  |  |  |  |
| No | 1.000 |  | 1.000 |  | 0.844 (0.713, 0.999) | **0.048** |
| Yes | 0.301 (0.270, 0.336) | **<0.001** | 0.436 (0.353, 0.539) | **<0.001** | 1.206 (1.020, 1.425) | **0.028** |
| Chemotherapy |  |  |  |  |  |  |
| No | 1.000 |  | 1.000 |  | 1.115 (0.912, 1.364) | 0.288 |
| Yes | 0.983 (0.853, 1.131) | 0.806 | 0.877 (0.667, 1.154) | 0.348 | 1.017 (0.806, 1.283) | 0.886 |
| Radiotherapy |  |  |  |  |  |  |
| No | 1.000 |  | 1.000 |  | 1.059 (0.895, 1.252) | 0.506 |
| Yes | 1.219 (0.906, 1.639) | 0.190 | 1.777 (0.966, 3.271) | 0.065 | 1.625 (0.842, 3.135) | 0.148 |
| Palliative |  |  |  |  |  |  |
| No | 1.000 |  | 1.000 |  | 1.099 (0.968, 1.247) | 0.146 |
| Yes | 2.961 (2.539, 3.454) | **<0.001** | 2.148 (1.555, 2.967) | **<0.001** | 0.830 (0.594, 1.159) | 0.274 |
| Hp |  |  |  |  |  |  |
| Negative | 1.000 |  | 1.000 |  | 0.989 (0.702, 1.394) | 0.950 |
| Positive | 1.493 (1.117, 1.996) | **0.007** | 1.665 (0.891, 3.111) | 0.110 | 0.869 (0.479, 1.579) | 0.646 |
| Ki67 |  |  |  |  |  |  |
| Low-expression | 1.000 |  | 1.000 |  | 1.204 (0.852, 1.701) | 0.293 |
| High-expression | 1.149 (0.944, 1.398) | 0.166 | 1.204 (0.835, 1.736) | 0.320 | 1.265 (1.005, 1.591) | **0.043** |
| VEGF |  |  |  |  |  |  |
| Low-expression | 1.000 |  | 1.000 |  | 1.065 (0.698, 1.622) | 0.771 |
| High-expression | 0.906 (0.724, 1.135) | 0.392 | 1.198 (0.779, 1.844) | 0.411 | 1.401 (1.100, 1.784) | **0.006** |
| EGFR |  |  |  |  |  |  |
| Low-expression | 1.000 |  | 1.000 |  | 1.175 (0.801, 1.724) | 0.410 |
| High-expression | 1.049 (0.834, 1.319) | 0.683 | 1.189 (0.799, 1.769) | 0.394 | 1.364 (1.063, 1.751) | **0.015** |
| GC, gastric cancer; *P* for intra-group univariate survival analysis; *P*a for inter-group univariate survival analysis. | | | | | | |
